# Effect of Nanoemulsion Carbonated Hydroxyapatite-Statin Administration on the Alkaline Phosphatase Level during Orthodontic Relapse in Rats 

**DOI:** 10.30476/dentjods.2025.103152.2426

**Published:** 2025-09-01

**Authors:** Lina Lestia, Niswati Fathmah Rosyida, Yanuarti Retnaningrum, Ananto Ali Alhasyimi

**Affiliations:** 1 Orthodontics Resident, Faculty of Dentistry, Universitas Gadjah Mada, Yogyakarta, Indonesia.; 2 Dept. of Orthodontics, Faculty of Dentistry, Universitas Gadjah Mada, Yogyakarta, Indonesia.

**Keywords:** Bone remodeling, Orthodontic, Simvastatin, Health

## Abstract

**Background:**

Relapse is acknowledged as a substantial failure after orthodontic correction. As a biomarker of bone formation, alkaline phosphatase (ALP) levels will decline in compressive sites during relapse subsequent to orthodontic tooth displacement. Manipulating alveolar bone remodeling to elevate ALP levels using novel biomaterial is presently regarded as one of the innovative approaches to avert relapse effectively.

**Purpose:**

This study aimed to evaluate the effect of carbonated hydroxyapatite (CHA)-statin nanoemulsion on ALP levels in relapsed rats.

**Materials and Method:**

In this *in vivo* study, forty-eight (*n*= 48) rats were divided into four groups: control, CHA, statin, and CHA-statin, with 12 rats in each group. A 30g mesial tract-ion was applied for seven days using a closed-coil spring extending from the first maxillary molar to the maxillary incisor. Over seven days, CHA, statin hydrogel, and nanoemulsion CHA-statin were injected every three days to preserve the repositioned teeth. Removing the appliances allowed for relapse to occur. ALP levels were assessed using an enzyme-linked immunosorbent assay on days 0, 1, 7, and 14 after debonding. The acquired data was analyzed using analysis of variance and a post hoc Tukey's test with a significance level of *p*< 0.05.

**Results:**

The average ALP levels between the groups did not differ significantly (*p*> 0.05) on days 0 and 1 of relapse. The mean values were highest in the CHA-statin nanoemulsion group. The control group exhibited the lowest average ALP levels on day 7 of the relapse phase, followed by Group CHA, Group statin (St), and Group CHA-St, with a significant difference (*p*< 0.05). On day 14, the CHA-St group had the highest average ALP levels (*p*< 0.05), while the CHA and St groups did not differ (*p*> 0.05).

**Conclusion:**

The results indicated that nanoemulsion CHA-statin could elevate ALP levels during orthodontic relapse

## Introduction

Orthodontics is a specialized field of dentistry that involves the diagnosis, prevention, and treatment of dental and maxillofacial disorders. Furthermore, this branch of dentistry aims to stimulate the development of the human jaw, face, and teeth through orthodontic treatment [ 1 ]. Ensuring stability in orthodontic treatment is a significant concern for both orthodontists and patients. Orthodontic relapse denotes the propensity of teeth to revert to their original positions after orthodontic intervention, and it is considered an undesirable outcome [ 2 ]. For this reason, good stable occlusion and occasionally overcorrection of malocclusions are necessary to obtain stable orthodontic treatment outcomes. However, previous study has identified a tendency to relapse in spite of this. Relapse in orthodontics is a complex and enigmatic phenomenon that is influenced by a variety of factors that undermine the stability of the results, including the timing of gingival and periodontal tissue reorganization [ 3 ]. The process of relapse following orthodontic treatment is identical to that of orthodontic tooth movement. During orthodontic movement, teeth move as a result of alveolar bone resorption caused by osteoclastic activity on the pressure side and bone deposition induced by osteoblastic induction on the tension side [ 4 ]. Bone resorption in the relapse direction will be induced by the positive change in the number of osteoclasts and their distribution along the alveolar bone, as well as the tooth adjacent to them [ 5 ]. Osteoblastogenesis and bone formation is believed to counteract osteoclastogenesis [ 6 ]. Prior research has established that the existence of alkaline phosphatase (ALP) in gingival crevicular fluid (GCF) functions as a biological marker for bone formation and remodeling during orthodontic tooth movement [ 7 ]. The escalating osteoblast activities that occur during the process of bone formation will be distinguished by the raising manifestations of ALP [ 8 ].

The implementation of a retainer is a common method for preventing orthodontic relapse. Popular removable appliances resembling Hawley are efficient at preserving the intended occlusion. In contrast, removable devices require increased patient cooperation and dependability regarding maintenance and utilization. In such cases, a fixed retainer might be a more appropriate alternative [ 9 ]. In orthodontic retention, fixed retainers are frequently utilized due to their minimal patient involvement, high effectiveness, and aesthetic appeal. Due to their difficult bonding requirements and potential to adversely affect periodontal health, their application is restricted [ 10 ]. Pharmacologically, bisphosphonates and analogous medications also exhibit an additive effect in the prevention of orthodontic relapse. Nevertheless, patients experience detrimental effects when exposed to bisphosphonates [ 11 ].

As of now, tissue engineering approaches have been proposed for the modulation of alveolar bone remodeling, the mitigation of orthodontic relapse, and the augmentation of tooth position stability. Significant potential exists for carbonate apatite (CHA) in the domain of bone tissue engineering on account of its capacity to regulate calcium release and promote bone formation [ 12 ]. CHA is regarded as an exceptional biomaterial for facilitating alveolar bone remodeling due to its structural resemblance to the porous and interconnected architecture of bone [ 13 ]. In recent times, there has been speculation regarding the potential therapeutic utility of simvastatin, a hypolipidemic agent, in the treatment of osteoporosis. These medications, referred to as statins, embody an innovative strategy in the management of osteoporosis by placing emphasis on the regeneration of bone tissue that has been compromised [ 14 ]. A previous study found that systemic simvastatin administration can reduce post-orthodontic relapse by reducing osteoclast-mediated bone resorption and promoting bone growth [ 15 ]. Furthermore, the exceptional long-term safety profile of simvastatin makes it a viable option for use into orthodontic treatment [ 16 ]. Large concentrations are necessary to induce bone effects from statins, which can potentially lead to increased toxicity and adverse effects, including rhabdomyolysis [ 17 ]. By administering simvastatin locally, one can mitigate its hepatotoxic properties and enhance its effectiveness in promoting bone repair [ 18 ]. As potential drug delivery systems, nanoemulsions have gained traction, serving as both topical administration systems and bioavailability enhancers for active pharmacological components that are inadequately soluble in water [ 19 ]. This study was conducted to evaluate whether the use of nanoemulsion CHA-statin may enhance ALP levels in rats experiencing experimental relapse. 

## Materials and Method

The animal experimentation adhered to the protocols established in the National Institutes of Health Guide for the Care and Use of Laboratory Animals. The Research Ethics Committee of the Faculty of Dentistry, UGM, approved item number 41/UN1/KEP/FKG-RSGM/EC/ 2023. Forty-eight male *Sprague-Dawley* rats, each weighing 250±25g at eight weeks of age, were used. The rats were randomly assigned to four independent groups: group C (control, no treatment); group St (statin nanoemulsion); group CHA (CHA hydrogel); and group CHA-St (a combination of nanoemulsion CHA and statin). A random subset of four subgroups (*n*= 3) was formed from each group based on the day of observation. For each experiment, anesthetized rats were used. Prior to the initiation of the experiments, a week of acclimatization was undertaken to facilitate the animals' adjustment to their new surroundings and laboratory diet. 

To produce CHA hydrogel, a solution was prepared by combining type-I gelatin, distilled water, and sodium citrate. After incorporating calcium hydroxide, the resulting solution was subjected to magnetic stirring for one hour. Phosphoric acid was mixed in 50 mL of water before being added to the gelatin mixture, which was poured with precaution. After synthesis, a solution was prepared by combining 100μL of the nanoemulsion statin with 10 mg of CHA that had been autoclaved at 105°C for 60 minutes. Following this, the hydrogel was incubated for one hour at 37°C to promote electrostatic binding. Following the synthesis process, 50mg of CHA was amalgamated with 5mL of distilled water to generate the hydrogel CHA. A gel comprising simvastatin nanoemulsion was made by combining 100 µl of oleic acid, 400µl of Tween 80, 25mg of simvastatin, and 2ml of distilled water with a gel base consisting of 25 mg of carbomer and 2.5 ml of hot water in equal ratios. The CHA-simvastatin nanoemulsion gel was developed by mixing simvastatin nanoemulsion gel (0.05mg/10µl) with CHA (0.1mg/10µl) in a 1:1 ratio.

In order to produce anesthesia in rats, an intramuscular infusion of a solution containing ketamine and Xylazine (with a dose of 35 mg/kg BW; 5 mg/kg BW) was given to the rats. A NiTi coil spring 6 mm (704-6065, Ormco, Orange, United States) had been attached following anesthesia, applying a split-mouth design. During the installation procedure, one end of the spring was situated between the right maxillary first molar and the incisor. To facilitate the ligature wire, the distal end of the spring was secured to the maxillary incisor by forming a groove in both incisors with a round drill. Ligature wire was threaded interdentally between the first and second molars and twisted with a clamp until it fitted to the grooves. The first molar was displaced mesially with the orthodontic appliance, while the second and third molars were anchored. The dynamometer calibration revealed an initial force of 30 grams force (MedKraft Orthodontics, USA). The closed-coil spring serving as a retainer was covered with flowable composites to render it inactive. The stabilization period was seven days, during which the distance was maintained at ± 2 mm. The molars commenced relapsing movement following the stabilization period, subsequent to the removal of both the wire and closed-coil spring. In the distal side of the gingival sulcus of the incisors, the treatment groups were administered 15 µl of CHA hydrogel (CHA group), nanoemulsion statin (St group), and nanoemulsion CHA-statin (CHA-St group) intrasulcularly on days 0, 4, and 7 of the stabilization period under general anesthesia. 

Following the collection of GCF samples, moderate airflow was utilized to dehydrate the gingival sulcus of each rodent. On days 0, 1, 7, and 14, GCF samples were alternatingly collected using a #20 paper point throughout the relapse phase. At a depth of approximately 1 mm, the paper point was cautiously placed into the gingival sulcus of the distal molar. The object remained stationary for a period of sixty seconds. Following that, two submerged paper points were transferred into a 1.5 mL Eppendorf tube containing 350 L of physiological saline solution. Enzyme-linked immunosorbent assay was employed to quantify ALP concentrations throughout the course of relapse. For the analysis, the quantitative anti-ALP antibody-specific reagent (Abcam®) was utilized. The total quantity of the transcription factor was compared to the value of its standard curve. Using a microplate reader, the optical density of the solutions was assessed at 450 nanometers. The notation used to represent the cumulative concentration of ALP was picograms per milliliter (pg/mL).

The statistical analysis of the data obtained in this study was conducted using two-way analysis of variance to identify differences and interactions between groups. Following that, statistically significant distinctions between groups were determined utilizing Tukey's honest significant difference test. It was considered that a p value less than 0.05 signified statistical significance. 

## Results

**Figure 1:**
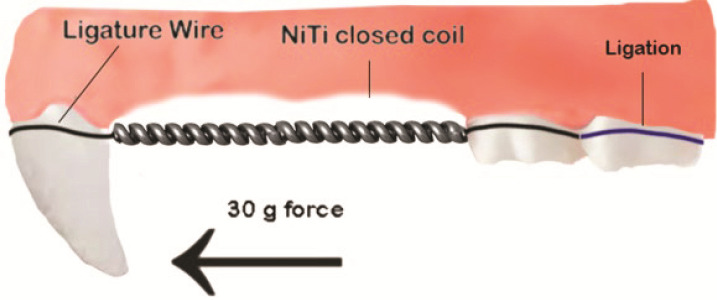
The design of a rat model for experimental orthodontic tooth movement

**Figure 2:**
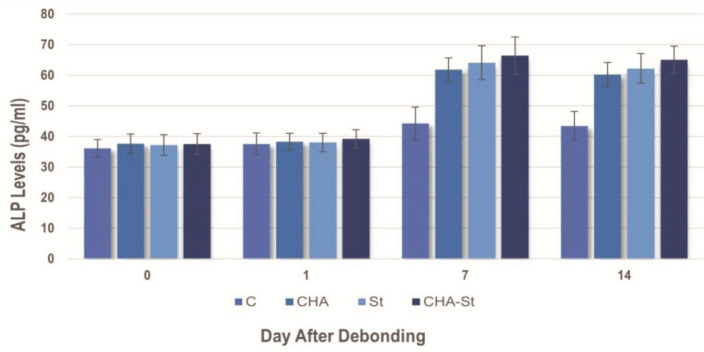
Mean (standard deviation) of alkaline phosphatase levels (picograms per milliliter; pg/ml) from the four groups examined. C, the control group; CHA, CHA group; St, statin group; CHA-St, CHA-statin group

The animals did not exhibit any indications of distress during the course of the experiments. The levels of ALP varied among the groups. Table 1 and Figures 1-2 shows the mean value of ALP levels measured in GCF using enzyme-linked immunosorbent assay. The normality and homogeneity test revealed that the data was normally distributed and homogeneous (*p*> 0.05). The two-way analysis of variance showed a significant rise in ALP levels. The mean of all groups increased significantly (*p*< 0.05) from day 1 to day 7. On the seventh day of the relapse phase, the control group exhibited the lowest average, followed by Group CHA, Group St, and Group CHA-St. Nevertheless, the group that received statin and CHA-statin nanoemulsion did not exhibit any difference in the elevation of ALP levels throughout orthodontic relapse on day 7 (*p*> 0.05).

**Table 1:** Comparison of alkaline phosphatase levels (picograms per milliliter) at each observation time point among the 4 tested groups

Parameter	C	CHA	St	CHA-St	*p* value	Post hoc comparison
ALP level						
Day 0	36.10 ± 2.86	37.61 ± 3.55	37.17 ± 5.37	37.52 ± 4.71	0.098	NS
Day 1	37.55 ± 3.22	38.25 ± 2.71	38.01 ± 3.86	39.19 ± 4.01	0.087	NS
Day 7	44.24 ± 3.36	61.83 ± 2.96	64.11 ± 5.49	66.42 ± 4.81	0.000*	C<CHA<St,CHA-St
Day 14	43.47 ± 3.42	60.19 ± 3.02	62.19 ± 6.13	65.07 ± 4.42	0.001*	C<St,CHA<CHA-St

The values are displayed using the mean along with the standard deviation.

Tested by two-way analysis of variance and post hoc Tukey’s honest significant difference test; C, the control group; CHA, CHA group; St, statin group; CHA-St, CHA-statin group.

ALP, Alkaline phosphatase

* *p* < .05, significant difference between groups; NS, not significant

There was no significant difference (*p*> 0.05) in the average ALP levels between the groups on days 0 and 1 of orthodontic relapse movement. Even so, the group that received CHA-statin nanoemulsion exhibited the highest mean values among all groups. Between days 7 and 14, all groups experienced a drop in ALP levels. On day 14, there was a significant mean difference (*p*< 0.05) between the highest averages of groups CHA-St, CHA, St, and the control group. Nevertheless, the group that received statin nanoemulsion and CHA hydrogel did not exhibit any difference in the elevation of ALP levels during relapse on day 14 (*p*> 0.05). 

## Discussion

**Figure 3:**
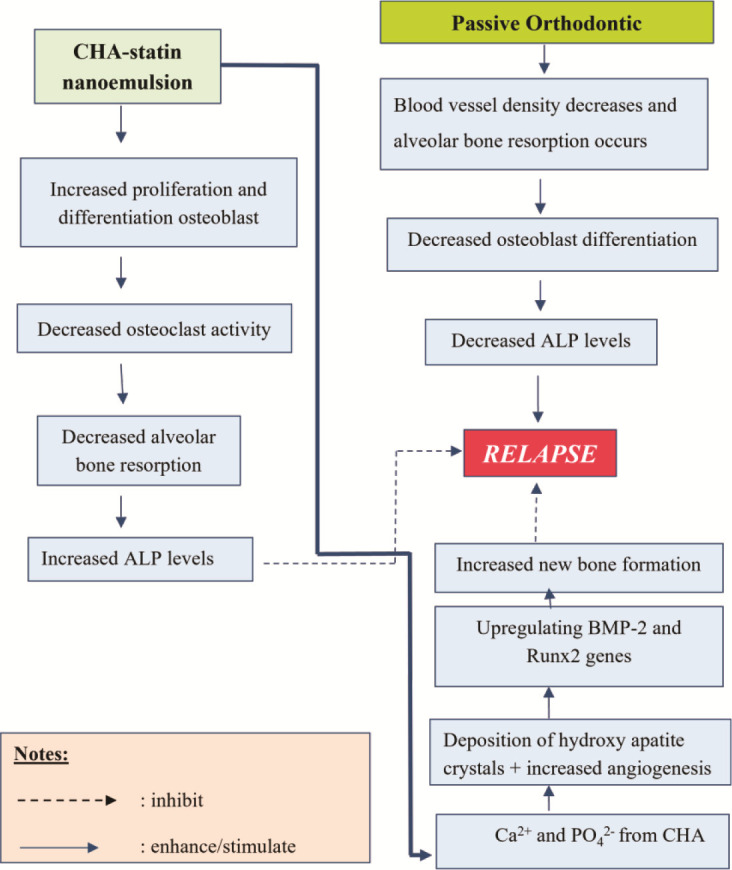
A brief outline of the method through which nanoemulsion CHA-statin enhances alkaline phosphatase levels. BMP-2, bone morphogenetic protein-2; Runx2, runt-related transcription factor 2; Ca^2+^, calcium; PO_4_^2-^, phosphate

This study showed that applying nanoemulsion CHA-statin significantly elevated ALP levels during relapse movement in rats compared to other groups. The potential of CHA to serve as a drug delivery system for the transportation of proteins into living cells has also generated considerable interest [ 20 ]. The biocompatibility and bioactivity of CHA are improved by the addition of carbonate ions to hydroxyapatite. CHA improves bone remodeling by elevating the levels of calcium and phosphate in the local environment, which are essential for forming new bone [ 5 ]. Osteoblast function is regulated by the discharge of calcium and phosphate ions into the surrounding tissue. Additionally, osteoblastic cells are stimulated to synthesize DNA and chemotaxis by elevated extracellular calcium levels, while osteoclastic formation is inhibited [ 5 ]. The augmentation of osteoblast activity during bone formation would be accompanied by the escalation in the expression of enzyme ALP [ 13 ]. This finding is also in agreement with a previous study, which showed that simvastatin significantly and promptly affected bone formation in osteoblasts by enhancing ALP activity [ 21 ]. Statins promote osteoblastic differentiation by upregulating the bone morphogenetic protein-2 (BMP-2) and runt-related transcription factor 2 (Runx2) genes and blocking the activity of the glucocorticoid receptor [ 22 ]. BMP-2 stimulates the transcription of the osteoblast-specific osteocalcin gene by activating Runx2, a critical transcription factor associated with osteoblast differentiation. This process results in the formation of new bone. Statins may enhance the transcriptional activity of Runx2 by increasing its expression, which results in the differentiation of mesenchymal stem cells (MSCs) into osteoblastic cells [ 22 ]. In the presence of an appropriate environment and stimuli, MSCs can facilitate the differentiation of osteoblasts. Although distinct contrasting characteristics have been identified, there are no definitive markers for osteogenic lineage cells. MSCs express specific genes, including ALP, osteopontin, osteocalcin, and type 1 collagen, during osteogenic differentiation [ 23 ]. Osteogenic differentiation is suggested by an increase in the expression of these markers. ALP activity initially decreases during the development of new bone by the osteoprogenitor, but it subsequently increases upon the occurrence of bone matrix differentiation and maturation [ 23 ]. Additionally, the nanoemulsion formulation employed in this study can potentially improve the biological activity of encapsulated pharmaceuticals *in vitro* and *in vivo* by enhancing these drugs' stability and physicochemical properties [ 19 ]. Figure 3 presents a concise overview of the mechanism by which nanoemulsion CHA-statin increases ALP levels.

The results of this study did not indicate any substantial changes in the levels of ALP on day 0 and day 1 of the study period. In the initial phase, bone resorption is more prevalent than bone deposition; however, both bone resorption and deposition occur simultaneously in the subsequent phase [ 24 ]. Hyalinization of the periodontal ligament may result from the disruption of blood flow caused by gingival and periodontal tissue fiber tension during alveolar bone formation. Undermining resorption was identified and relapse ensued when the tooth was observed for an extended period [ 25 ]. In contrast, significant differences were observed in each group on days 7 and 14. Alhasyimi *et al*. [ 13 ] conducted a study in which ALP levels increased on days 7 and 14 post-stabilization following orthodontic relapse in rabbits. This result aligns with the findings of the current investigation. ALP levels in gingival fibroblasts (GCF) can indicate tissue changes that occur during orthodontic tooth movement in periodontal tissues. Enhanced ALP enzyme expression is correlated with increased osteoblast activity during bone formation, which indicates an increase in the formation of new bone tissue. It is well-established that the process of bone remodeling, which began with initial resorption activity, took place on days 3-5 and was repeated on days 5-7. The final bone deposition occurred in both the pressure and tension locations on the walls of the alveolar bone on days 7-14 [ 26 - 27 ]. Our investigation is limited by the fact that we have selected the ALP biomarker for osteoblast activity labelling via ELISA, rather than relying on visual analysis to directly define osteoblasts. The method of counting bone formation activity may have been somewhat subjective. Consequently, an additional investigation utilizing the immunohistochemistry approach to evaluate biomarkers of osteoblast activity (including osteocalcin, procollagen type 1 N-terminal propeptide (P1NP), and procollagen type 1 C-terminal propeptide (P1CP)) is required to strengthen this study. 

## Conclusion

The results indicated that the administration of nanoemulsion CHA-statin may result in an elevation in ALP levels following orthodontic relapse. This finding has the potential for translation into clinical settings as a nanoemulsion CHA-statin functions as an effective agent to diminish orthodontic relapse. This study developed a minimally invasive, cost-effective method for the application of an osteoinductive and osteoconductive substance, which is effective in minimizing relapse following orthodontic tooth movement. Nevertheless, it is essential to perform more extensive clinical studies to further confirm and elucidate the effectiveness of nanoemulsion CHA-statin in humans.
